# A Self-Supervised Anomaly Detector of Fruits Based on Hyperspectral Imaging

**DOI:** 10.3390/foods12142669

**Published:** 2023-07-11

**Authors:** Yisen Liu, Songbin Zhou, Zhiyong Wan, Zefan Qiu, Lulu Zhao, Kunkun Pang, Chang Li, Zexuan Yin

**Affiliations:** 1 Institute of Intelligent Manufacturing, Guangdong Academy of Sciences, Guangzhou 510070, China; ys.liu@giim.ac.cn (Y.L.); zy.wan@giim.ac.cn (Z.W.); zf.qiu@giim.ac.cn (Z.Q.); 2 Guangdong Key Laboratory of Modern Control Technology, Guangzhou 510070, China; ll.zhao@giim.ac.cn (L.Z.); kk.pang@giim.ac.cn (K.P.); c.li@giim.ac.cn (C.L.); zx.yin@giim.ac.cn (Z.Y.)

**Keywords:** defect detection, fruit quality control, near-infrared hyperspectral imaging, self-supervised learning

## Abstract

Hyperspectral imaging combined with chemometric approaches is proven to be a powerful tool for the quality evaluation and control of fruits. In fruit defect-detection scenarios, developing an unsupervised anomaly detection framework is vital, as defect sample preparation is labor-intensive and time-consuming, especially for exploring potential defects. In this paper, a spectral–spatial, information-based, self-supervised anomaly detection (SSAD) approach is proposed. During training, an auxiliary classifier is proposed to identify the projection axes of principal component (PC) images that were transformed from the hyperspectral data cubes. In test time, the fully connected layer of the learned classifier was used as a ‘spectral–spatial’ feature extractor, and the feature similarity metric was adopted as the score function for the downstream anomaly evaluation task. The proposed network was evaluated with two fruit data sets: a strawberry data set with bruised, infected, chilling-injured, and contaminated test samples and a blueberry data set with bruised, infected, chilling-injured, and wrinkled samples as anomalies. The results show that the SSAD yielded the best anomaly detection performance (AUC = 0.923 on average) over the baseline methods, and the visualization results further confirmed its advantage in extracting effective ‘spectral–spatial’ latent representation. Moreover, the robustness of SSAD is verified with the data pollution experiment; it performed significantly better than the baselines when a portion of anomalous samples was involved in the training process.

## 1. Introduction

Hyperspectral imaging combined with chemometric approaches is proven to be a powerful tool for the quality evaluation and control of fruits [1,2] as it enables the assessment of internal properties that cannot be inspected with computer vision, including soluble solid content [3], acidity [4], and texture [5]. Detecting fruit quality defects, such as unripeness [6], bruise [7], contamination [8], chilling injury [9], fungal infection [10], and skin defects [11], is also of great importance since these defects make the fruits less attractive to the consumers or even bring food safety risks. Quite a few efforts have been made on chemometric algorithms to build fruit defect detectors. For instance, Liu et al. [12] selected optimal wavelengths with successive projection algorithms (SPA) and compared partial least square discriminant analysis (PLS-DA), support vector machine (SVM), and back-propagation neural network (BPNN) algorithms to identify bruised and fungi-infected strawberries. Tian et al. [13] established citrus decay detection models by using two-band ratio images and improving watershed segmentation with a total success rate of 92% at the image level. G. ElMasry et al. [14] detected chilling injury in red delicious apples using 7hyperspectral imaging and adopted the artificial neural network (ANN) to select the optimal wavelengths, classify the apples, and detect firmness changes due to chilling injury.

In the past few years, the impressive performance of various deep-learning applications has also appealed to the chemometric community, and some deep detectors of fruits have been developed. Zhang et al. [15] detected blueberry internal bruises from hyperspectral transmittance images using fully convolutional networks. In our previous work [16], strawberry bruises were identified with a two-branch convolutional network in which the one-dimensional branch and the two-dimensional branch extracted the spectral and spatial information from the hyperspectral data, respectively. However, both the conventional machine-learning approaches and the deep-learning-based methods mentioned above built supervised classification models that require collecting a large number of targeted anomalous fruits as training data for each defect class. Developing effective unsupervised anomaly detection methods for fruit quality control is crucial, as the defect sample preparation could be labor-intensive and time-consuming. Most importantly, exploring all defects exhaustively in advance is impractical. Anomaly detection techniques are able to identify all unknown defects by only training with normal samples, which shows great potential for applications of intelligent fruit sorting.

Unsupervised anomaly detection [17,18], also known as novelty detection or out-of-distribution detection, has made great progress and has been successfully applied to image, sound, video, and medical data. The existing methods for this task can generally be grouped into four categories: reconstruction-based methods, clustering-based methods, classification-based methods, and self-supervised methods. Autoencoder (AE) [19] is the most widely used reconstruction-based method as it works based on the assumption that anomalous samples suffer from larger reconstruction costs, while the low-dimensional projection functions are only trained with the normal samples. The general idea of clustering-based methods is to learn the distribution of the normal samples and that the anomalous test samples lie in the low-density regions, such as Gaussian Mixture Models (GMM) [20] and Local Outlier Factor (LOF) [21]. Regarding the classification-based methods, one-class SVM (OCSVM) [22] is the most well-known algorithm, which tries to learn a classification boundary surrounding the normal samples in the feature space.

An anomaly detector based on self-supervised learning [23,24] is a new emerged category in which auxiliary tasks are designed to learn informative, low-dimensional representations from normal data, and the learned representations are then used for the downstream anomaly detection task. For instance, Golan et al. [25] designed an auxiliary geometric transformation classifier to detect image anomalies. Zhang et al. [26] generated auxiliary data from various distributions and built an auxiliary classifier to discriminate the real normal data from the generated data. Then, a deep adversarial training model was constructed to capture marginal distributions of the normal data in the latent space learned with the classifier. Tack et al. [27] combined contrastive learning and a shifted-instance classification task to construct anomaly scores. In general, recent studies show that self-supervised learning can easily integrate with various deep-learning frameworks and significantly help to extract discriminative features for anomaly detection, especially for machine vision tasks.

Despite anomaly detection’s success in benchmark data sets of natural images, work on anomaly detection of hyperspectral imaging data is generally lacking. One of the main challenges for anomaly detection of hyperspectral data is capturing anomalies with the integration information of both the spectral and spatial domains. Most of the anomaly detection works in the chemometric community were conducted on near-infrared (NIR) spectroscopy or Raman spectroscopy without spatial information involved [28,29,30]. For instance, Vasafi et al. [31] adopted AE for milk anomaly detection based on NIR spectroscopy and discussed the effect of NIR spectral pretreatments. Shen et al. [32] used the global H value discrimination method and NIR spectroscopy to detect non-protein nitrogen adulterations in soybean meal. Only spectral information was considered in these approaches, and anomaly detection frameworks based on ‘spectral–spatial’ fusion remain to be explored.

Based on these concerns, we present a simple yet effective self-supervised anomaly detector (referred to as SSAD) for fruit defect detection. In the proposed framework, the hyperspectral data cubes were transformed into principal component (PC) images, and an auxiliary classifier was designed to classify the PC number of these images. Discriminative features for anomaly detection were extracted with the auxiliary PC image classifier, and the feature similarity metric was adopted for anomaly evaluation. The PC images retain the spatial features of the fruits, and the grayscale of each pixel reflects its spectral characteristics. Therefore, by constructing a PC image classifier, efficient representations that integrate the information of the spectral and spatial domains can be learned and utilized for anomaly detection. To the best of our knowledge, we are the first to demonstrate the ability to integrate the spectral–spatial information of hyperspectral data under the anomaly detection framework.

The proposed method was respectively evaluated in strawberry and blueberry data sets to demonstrate the effectiveness and versatility of this self-supervised anomaly detection approach. For each data set, four quality defect types were utilized to validate the ability of SSAD in identifying multiple hyperspectral anomalies. To be specific, the strawberry data set tested bruised, fungal-infected, chilling-injured, and soil-contaminated samples, while the blueberry data set used bruised, fungal-infected, chilling-injured, and wrinkled samples as anomalies. For performance comparison, three spectral models, including OCSVM, one-dimensional AE (AE-1D) and one-dimensional variational AE (VAE-1D), and two ‘spectral–spatial’ models with PC images as inputs, namely two-dimensional AE (AE-2D) and two-dimensional variational AE (VAE-2D), were also constructed in the present work.

## 2. Materials and Methods

### 2.1. Samples

Two fruit hyperspectral data sets were prepared to validate the proposed anomaly detection method, namely the strawberry and the blueberry data sets.

#### 2.1.1. Strawberry Data Set

For the strawberry data set [12,16], a total of 1045 strawberry samples were measured, including 601 normal and 444 anomalous samples. Four types of anomalies for the strawberries are defined: bruise, fungal infection, chilling injury, and soil contamination. All the strawberry samples were purchased from local supermarkets in Guangzhou, China. The strawberry samples included three varieties (Hongyan, Hongbaoshi, Shuangliu, and Zhangji) and went through a visual check for healthy appearance during the purchase.

Bruised strawberries: A total of 139 bruised strawberries were obtained using mechanical vibration and pressure to simulate the damage during packaging and transportation. The hyperspectral image measurements were conducted within 30 min after the mechanical injury. 

Infected strawberries: A total of 100 fungal-infected strawberries were prepared by injecting the Botrytis cinerea spore solution. The solution concentration was 1 × 10^5^ CFU∙mL^−1^, and the injection depth was 2 mm. Hyperspectral image measurements were carried out every 12 h during a storage period of 24 to 84 h after the injection.

Chilling-injured strawberries: A total of 105 chilling-injured strawberries were prepared by keeping the strawberry samples in cold storage at −1 °C for 0.5~6 h. The injured strawberries were removed from the cold storage and left at room temperature for another 4 h to regain their temperature before the measurements.

Contaminated strawberries: A total of 100 soil-contaminated strawberries were prepared by applying soil dilutions to the strawberries. The contaminated samples were dried in the air before the hyperspectral measurements.

#### 2.1.2. Blueberry Data Set

For the blueberry data set [15,33], a total of 1335 blueberry samples were measured, including 808 normal and 527 anomalous blueberries. All the blueberry samples were purchased from local supermarkets in Guangzhou, China. For the normal samples, a visual check was performed before the measurements to ensure they were free from any abnormal features. Four types of anomalies, including bruise, fungal infection, chilling injury, and wrinkled skin, were prepared as follows.

Bruised blueberries: A total of 120 bruised blueberries were obtained using mechanical vibration and pressure. The injured blueberries were stored for another 0.5~12 h before the hyperspectral image measurements to allow the injury development.

Infected blueberries: A total of 120 fungal-infected blueberries were prepared. Half of the infected blueberries were obtained by injecting the Botrytis cinerea spore solution, while the other half were obtained naturally by storage.

Chilling-injured blueberries: A total of 150 chilling-injured blueberries were prepared. The chilling injury of the blueberries was also obtained by freezing the samples at −1 °C for 0.5~6 h and returning them to room temperature for 4 h.

Wrinkled blueberries: A total of 137 wrinkled blueberries were obtained by storage. The blueberries were stored at room temperature for 3 to 8 days to obtain samples with wrinkled skin.

**Figure 1:**
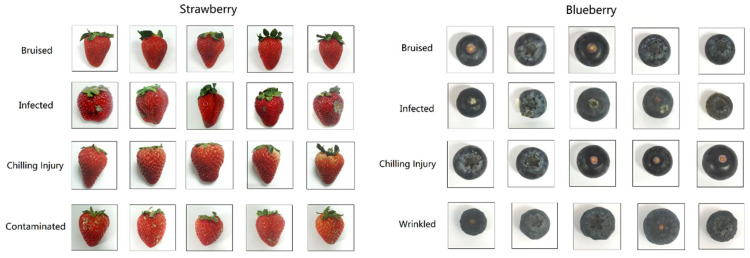
Photographs of the anomalous strawberry and blueberry samples.

In the training phase, half of the normal samples were randomly selected and used for building the models. Regarding the testing phase, the rest of the normal samples and all the anomalous samples were adopted for model evaluation. The sample partition of the strawberry and the blueberry data sets is summarized in Table 1, and the digital color photos of the anomalous strawberry and blueberry samples are shown in Figure 1. It can be observed that the bruise and the chilling injury are difficult to identify visually.

### 2.2. Hyperspectral Data Measurement

**Figure 2:**
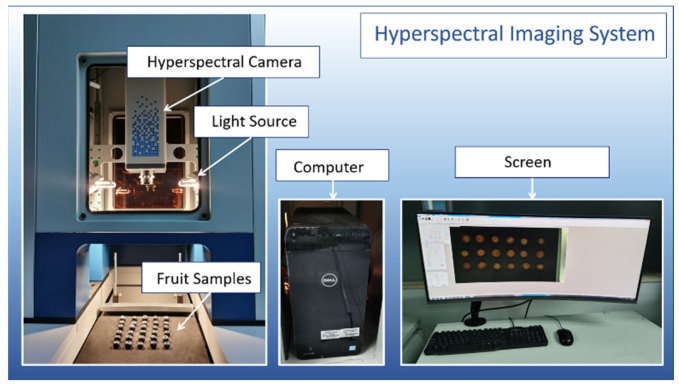
Hyperspectral imaging system.

The hyperspectral data of the strawberry and blueberry samples were measured with a NIR hyperspectral imaging instrument (SPECIM, Spectral Imaging Ltd. Oulu, Finland). The hyperspectral imaging system worked with the diffraction grating and the InGaAs sensor matrix. The focal length of the optical lens was 30.7 mm. The fruit samples were placed on a holder plate and moved with the translation stage to form three-dimensional data cubes line by line (see Figure 2). For the strawberry data set, the samples were placed in random positions, while the blueberry samples included both stems and calyces upwards. Each hyperspectral image had 320 × 640 pixels in the spatial dimension and 256 wavelengths in the spectral dimension. The spatial resolution was approximately 0.4 mm/pixel in both the *x*-axis and *y*-axis [34], while the spectral resolution of the equipment was 5 nm. The wavelength range of the raw data was 885~1733 nm, but we deleted the initial and the terminal wavelength sections because they were poor in signal quality. Finally, wavelengths ranging from 1000 to 1600 nm (181 wavelengths) were used for modeling.

### 2.3. Data Preprocessing

The preprocessing converted the raw hyperspectral data into standard sample data cubes. There are three steps for preprocessing: image correction, first-order derivative, and image segmentation. The image correction step calculates the relative reflectance of each pixel by using a dark current image and a white reference image:

(1) $$I_{C}=\frac{I_{0}-I_{D}}{I_{w}-I_{D}}$$

where $I_{0}$ is the original image, $I_{D}$ is the dark image, $I_{w}$ is the white image, and $I_{c}$ is the corrected image. First-order Savitzky–Golay derivative was performed on the spectrum of each pixel for pretreatment. Image segmentation was performed with the watershed algorithm [35] to remove the background and extract the sample data cubes from the whole hyperspectral image. The extracted pixels of each fruit sample were placed in the center of a blank background. The size of the data cube for the strawberry data set was 120 × 120 × 181, while it was 60 × 60 × 181 for the blueberry data set.

### 2.4. The Proposed Method

#### 2.4.1. Architecture of SSAD

**Figure 3:**
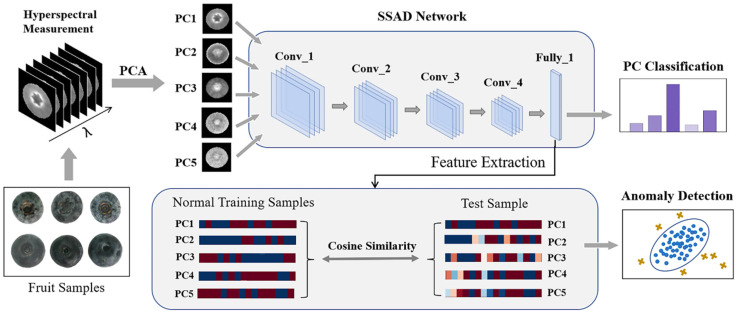
The architecture of the proposed SSAD for fruit anomaly detection.

As shown in Figure 3, the proposed SSAD model was constructed as follows: (1) Performed PCA transformation, which changed the sample-scale data cube into the first *m* PC images $\left[x_{1}^{\prime },x_{2}^{\prime },x_{3}^{\prime },\ldots {,x}_{m}^{\prime }\right]$; (2) constructed the auxiliary PC image classifier by training it to identify the PC number of the normal fruit samples; (3) used the trained fully connected layer of the auxiliary classifier as a ‘spectral–spatial’ feature extractor of the first *m* PC images ($F=[F_{pc1},F_{pc2},F_{pc3},\ldots ,F_{pcm}]$); and (4) in the testing phase, adopted the cosine similarity $S\left(x_{t}\right)$ to the training set in the obtained feature space for anomaly evaluation of the unknown samples.

#### 2.4.2. Detailed Description of Training Procedure

In the training phase, the PC images transformed from the normal samples were fed into the convolutional network to train an auxiliary PC classifier. This classifier was stacked with convolution layers, pooling layers, and a fully connected layer. In the convolution layer, the feature maps of the previous layer were convolved with learnable convolutional filters and processed with the nonlinear activation. The operation of the *l*’th convolution layer was:

(2) $$F^{l}=g\left(F^{l-1}*w^{l}+b^{l}\right)$$

where * represents the convolution operation, $F^{l-1}$ refers to the feature map obtained with the previous layer, and $F^{l}$ presents the feature map of the current layer, $w^{l}$ and $b^{l}$ are the trainable weight and bias of the *l*’th convolution layer, respectively, and g(x) is the nonlinear activation of the convolution layer.

The fully connected layer was adopted to integrate the local information obtained with the previous convolution layers and pooling layers. The operation of the fully connected layer can be presented as:

(3) $${\;F}_{i}^{f}=\sigma \left(\sum_{j=1}^{k}F_{j}^{f-1}\times w_{i,j}^{f}+b_{i}^{f}\right),i=1,2,3,\ldots ,n.\;$$

where $F_{i}^{f}(i=1,2,3,\ldots ,n)$ represents the output of the fully connected layer, *n* is the total neurons number of the fully connected layer, $F_{j}^{f-1}(j=1,2,3,\ldots ,k)$ represents the output of the previous layer, *k* is the feature map size of the previous layer, $w_{i,j}^{f}$ and $b_{i}^{f}$ represent the trainable weight and bias of the fully connected layer, respectively, and σ(x) is the nonlinear activation of the fully connected layer, which is tanh in this paper. Finally, a SoftMax regression layer followed the fully connected layer to provide the m-class probabilities result. The classifier was trained to minimize the cross-entropy loss:

(4) L=1N∑i=1Nyilog y^i

where *N* is the number of normal training samples, y is the ground truth of PC number, and $\hat{y}$ is the predicted probabilities.

#### 2.4.3. Detailed Description of Testing Procedure

In the testing phase, the feature extracted with the fully connected layer of the trained auxiliary PC classifier was used for constructing the anomaly scores. For each test sample, we fed the first *m* PC images into the classifier sequentially and concatenated the obtained features as the representation of one sample:

(5) $$F=[F_{pc1},F_{pc2},F_{pc3},\ldots ,F_{pcm}]$$

Upon the representation learned by our proposed training objective, we present the most effective score function: the mean cosine similarity to the training set in latent space

(6) $$S(x_{t})=-\frac{1}{N}\sum_{i=1}^{N}\frac{F_{t}\times F_{n}^{i}}{\sqrt{{\left(F_{t}\right)}^{2}\times {\left(F_{n}^{i}\right)}^{2}}}$$

where *S* represents the anomaly score, $x_{t}$ is the test sample, and $F_{t}$ and $F_{n}$ are the representations of the test sample and the normal training samples, respectively.

Instead of using a pre-chosen threshold, we followed the decision-making protocol of the state-of-the-art approaches to make normal/anomaly decisions. That is, sorting the anomaly scores of the test samples and those samples with the highest $N_{+}$ anomaly scores were assigned as anomalies, where $N_{+}$ was the number of anomalous samples in the test set.

#### 2.4.4. Hyperparameter Settings and Training Configurations

The hyperparameter *m* was assigned to 5 in the original SSAD, and its effect on anomaly detection is demonstrated in the discussion section. Cross-validation within the training data was performed to optimize the structural parameters of SSAD, including the number of convolution layers, the number of convolutional filters in each layer, and the number of pooling layers. It should be mentioned that the cross-validation was only processed to minimize the classification error of the validation set, and no data in the test set were involved. The details of the SSAD network parameters for the strawberry and blueberry data sets are listed in Table 2 and Table 3, respectively. It can be observed that we employed very similar network structures for the two data sets, only adding a pooling layer for the strawberry data set due to its larger input data size. For both data sets, the learning rate and batch size were set to 0.001 and 256, respectively. The training process was stopped when the training loss was smaller than 0.1 to prevent overfitting. The code implementation and learned models of SSAD are available at https://github.com/YisenLiu-Intelligent-Sensing/SSAD accessed on 18 May 2022.

### 2.5. Metrics for Model Evaluation

Several widely used metrics were adopted to measure the anomaly detection performance, including the area under the receiver operating characteristic curve (AUC), F_1_ score, and accuracy (Acc) of each normal/anomaly class. These metrics are defined as follows.

AUC was calculated with the anomaly scores of the test samples:

(7) $$\mathrm{AUC}=\frac{1}{N_{-}N_{+}}\sum_{i=1}^{N_{-}}\sum_{j=1}^{N_{+}}H(Score\left(x_{j}^{+}\right)-Score\left(x_{i}^{-}\right))$$

In Equation (10), $N_{-}$ and $N_{+}$ are the number of normal and the anomalous test samples, ${\left\{x_{i}^{-}\right\}}_{i=1}^{N_{-}}$ and ${\left\{x_{j}^{+}\right\}}_{j=1}^{N_{+}}$ are the normal and anomalous test samples, and H(a) is the hard-threshold function calculated according to the anomaly scores:

(8) $$H\left(a\right)=\left\{\begin{array}{c} 0\;\;if\;a\leq 0\\ 1\;\;if\;a>0 \end{array}\right.$$

Given the anomaly scores, the true positives (TP), false positives (FP), and false negatives (FN) were obtained based on the decision-making protocol mentioned above. Then, the F_1_ score was calculated as:

(9) $$F_{1}=\frac{2\times precision\times recall}{precision+recall}=\frac{2\times \frac{\mathrm{TP}}{\mathrm{TP}+\mathrm{FP}}\times \frac{\mathrm{TP}}{\mathrm{TP}+\mathrm{FN}}}{\frac{\mathrm{TP}}{\mathrm{TP}+\mathrm{FP}}+\frac{\mathrm{TP}}{\mathrm{TP}+\mathrm{FN}}}$$

It can be observed that the F_1_ score can be interpreted as a weighted average of precision and recall.

Acc is the accuracy of each class:

(10) $$\mathrm{Acc}=\frac{\mathrm{TP}}{\mathrm{TP}+\mathrm{FN}}$$

${\mathrm{Acc}}_{\mathrm{normal}}$, ${\mathrm{Acc}}_{\mathrm{bruised}}$, ${\mathrm{Acc}}_{\mathrm{infected}}$, ${\mathrm{Acc}}_{\mathrm{chilling}}$, ${\mathrm{Acc}}_{\mathrm{contaminated}}$, and ${\mathrm{Acc}}_{\mathrm{wrinkled}}$ represent the accuracies of the normal, bruised, infected, chilling-injured, contaminated, and wrinkled classes.

Ten resampling runs were carried out for each scenario, and the obtained average and 95% confidence intervals of the metrics were used for model evaluation. In each run, we randomly selected 50% of the normal data for training with the remaining 50% reserved for testing. Only data from the normal class were used for the training models, while both the normal and anomalous ones were taken for model evaluation.

### 2.6. Methods for Comparison

OCSVM [22]: The one-class support vector machine (OCSVM) tries to learn a hypersphere in the feature space that maps most of the training data into it. The spectra of effective pixels were averaged, and the obtained mean spectra were used as the inputs of the OCSVM models. The radial basis function (RBF) was adopted as the kernel function, and the boundary hyperparameter υ was searched in the range of [0.01, 0.02, 0.03, 0.04, 0.05, 0.1, 0.2, 0.3] to maximize the AUC.

AE-1D [31]: The AE-1D is a classical reconstruction-based method. In this paper, it was trained to compress and reconstruct the mean spectra, and the reconstruction errors obtained with the test samples were taken as the anomaly scores. Dense neural networks were adopted as the encoder and the decoder of the AE-1D. Cross-validation was performed to adjust its structural parameters and avoid overfitting.

VAE-1D [19]: The VAE-1D estimates the mean and variance parameters of the Gaussian distribution in the latent space and reconstructs the latent vectors sampled from the learned distribution. In this study, it employed mean spectra as the inputs and the reconstruction probability through Monte-Carlo sampling as the anomaly score. The VAE-1D kept consistent network architectures with the AE-1D for each data set, but it was trained with the combination of reconstruction residual loss and KL divergence loss.

AE-2D: The AE-2D was trained to compress and reconstruct the PC images of the hyperspectral data. For a fair comparison, the AE-2D used the same inputs (the first five PC images) as the SSAD. The reconstruction error of each PC image was calculated, respectively, and the maximum reconstruction error was taken as the anomaly score.

VAE-2D: The VAE-2D is the probabilistic graphical version of the AE-2D. It also utilized the PC images as the inputs and the reconstruction error as the anomaly score. Convolutional networks were employed for the AE-2D and the VAE-2D because such a network structure had proved to be effective in extracting latent representations of images. The network structures were optimized with cross-validation to ensure a good image reconstruction quality.

The detailed structural parameters of the autoencoder-based methods can be found in Tables S1–S3.

### 2.7. Software and Hardware Environment

In the present work, the SSAD and the AE-based methods were conducted on the open-source platform Pytorch, and the OCSVM models were performed in the machine-learning toolbox scikit-learn. All the experiments were conducted using a computer equipped with a single GeForce TITAN V GPU.

## 3. Results

### 3.1. Spectra and PC Images

**Figure 4:**
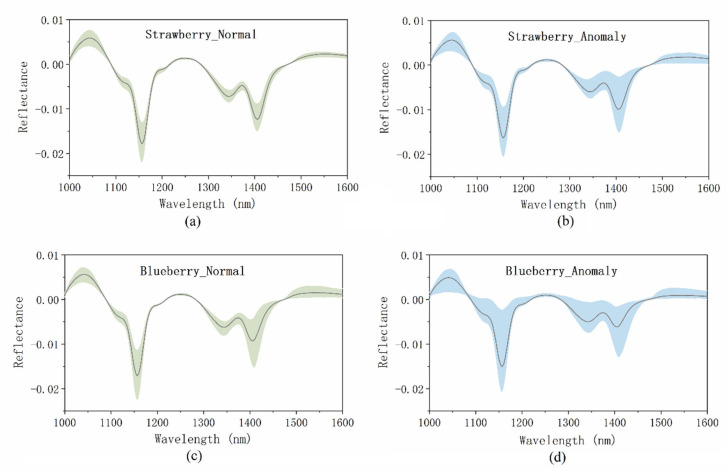
Spectral profiles of (**a**) normal strawberry samples, (**b**) anomalous strawberry samples, (**c**) normal blueberry samples, and (**d**) anomalous blueberry samples. Gray lines: mean spectral curves; colored areas: spectral profiles of all the samples.

To demonstrate the fruit anomalies in the spectral domain, the mean spectral profiles of the normal and anomalous samples in the strawberry and blueberry data sets are illustrated in Figure 4. The colored areas in Figure 4 represent the spectral profiles of all the samples to show their spectral distribution, while the gray lines are the mean spectral curves of the colored areas. For both data sets, the anomalous data had similar spectral profiles to the normal data. However, the anomalous samples demonstrated a wider distribution than the normal samples. For fruit samples, factors unrelated to quality defects, such as shape, variety, maturity, external and internal structure, etc., can also affect their mean spectra characteristics, which makes it difficult to detect quality anomalies from the spectral domain alone. The mean spectral profiles of each anomaly class are shown in Figure S1.

**Figure 5:**
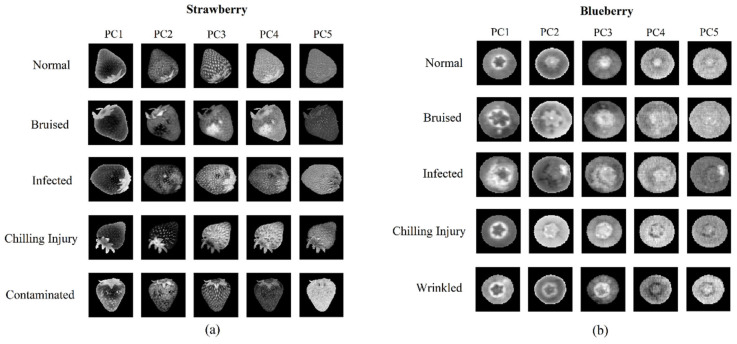
The first five PC images of the strawberry and blueberry data sets. (**a**) strawberry data set; (**b**) blueberry data set.

Motivated by detecting anomalies based on the combination of spectral and spatial domains, the SSAD extracted representation from the PC images. The first five PC images of the normal and anomalous fruits are demonstrated in Figure 5, while the images of PC6~PC10 can be found in Figure S2. It can be observed that, for some quality defects such as bruise, infection, and contamination, the anomalies appeared in the PC images as locally bright or dark areas. However, for the chilling injury, the anomaly appeared to be the overall grayscale change, as the freezing treatment caused the chemical and physical changes in the whole fruit. In general, the PC images were more informative for PC1 to PC7, whereas PC8 to PC10 lost a significant amount of detail because these PCs only accounted for a small fraction of the spectral variables. For more details of the variable explained ratios for the PCs, please refer to Figure S3.

### 3.2. Comparison of Anomaly Detection Performance

The anomaly detection results obtained with the SSAD and the baseline methods are illustrated in Table 4 and Table 5 for the strawberry and the blueberry sub-tasks, respectively. For each model, we performed ten resampling runs for the training set and test set partition, and the obtained average and 95% confidence intervals of the metrics are shown in the tables. It can be observed that the SSAD achieved the highest AUC and F_1_ score for both studied data sets. Compared with OCSVM, AE-1D, VAE-1D, AE-2D, and VAE-2D, the AUC improvements obtained with SSAD were 18.1%, 22.0%, 10.1%, 32.3%, and 38.5%, respectively, for the strawberry data set; when it comes to the blueberry data set, the AUC improvements with SSAD were 25.3%, 13.9%, 17.4%, 42.3%, and 16.1%, respectively. It should also be noticed that the SSAD obtained relatively balanced detection results (Acc >0.8 for each anomaly class), showing obvious superiority to both the autoencoder-based methods and the OCSVM.

In general, the spectral baseline models performed better than the ‘spectral–spatial’ baseline models: VAE-1D and AE-1D were the second-best performing methods for the strawberry and the blueberry data sets, respectively. As an anomaly detector, the autoencoder-based methods may suffer from the inefficiency of the extracted features because they are only trained to minimize the reconstruction error. For the AE-2D, in order to render good image reconstructions, it had to retain many fruit features irrelated to the quality defects, such as information about posture, shape, leaf, seed, calyx, stem, etc. On the other side, the auxiliary PC classification task of SSAD avoided most of these irrelated factors since they were unrelated to the class label (PC number). Therefore, more discriminative and robust ‘spectral–spatial’ features can be learned for the downstream anomaly detection task. We also listed the testing time per sample for each method in Table S4. The testing time of SSAD is less than one millisecond, which ranks second among all the comparison methods and is appropriate for the in-line inspection of a real application.

**Figure 6:**
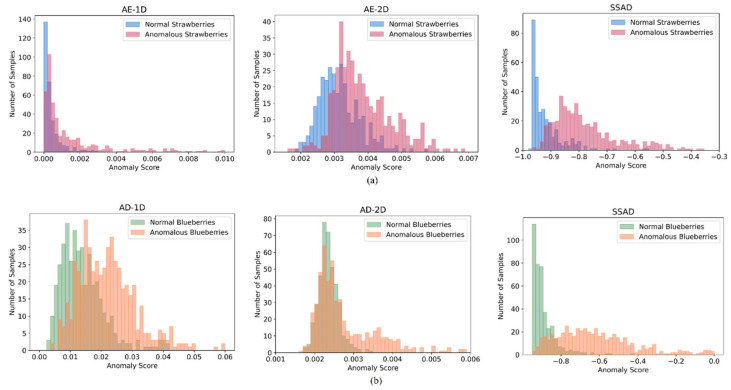
Anomaly score distributions obtained with the AE-1D, AE-2D, and SSAD: (**a**) strawberry data set; (**b**) blueberry data set.

Figure 6 compares the anomaly score distributions obtained with the AE-1D, AE-2D, and SSAD to visualize and compare their ability to distinguish between normal and anomaly. An ideal anomaly detector should be able to separate the anomaly scores of normal and anomalous samples. It can be observed that a significant proportion of anomalous test samples had the same level anomaly scores as the normal test samples under the AE-1D and AE-2D models, while the overlap was much smaller for the SSAD model. Moreover, the normal test samples demonstrated a narrow score distribution for the SSAD, which was advantageous for determining a decision threshold of normal/anomaly in the practical applications.

**Figure 7:**
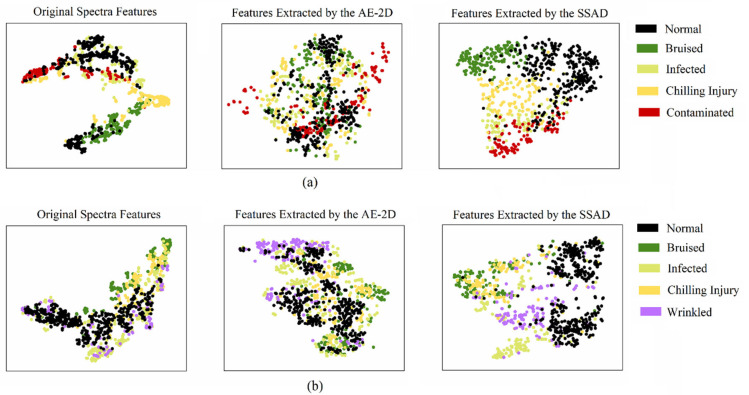
Visualization results of the original spectra, AE-2D features, and SSAD features by reducing the data dimension with the t-SNE algorithm: (**a**) strawberry data set; (**b**) blueberry data set.

To further verify the effectiveness of SSAD in learning discriminative representation for anomaly detection tasks, we demonstrate the feature visualization results obtained with the t-SNE algorithm [36] in Figure 7. It can be observed that the normal and anomalous points were highly overlapped in the latent space learned with AE-2D, indicating that the learned latent space was not task-specific for anomaly detection. In terms of the proposed SSAD, there were obvious clustering trends between the normal and anomalies data in the latent space, providing meaningful information to identify anomalies. It should be noticed that, although only normal samples were used for the self-supervised training, the unseen anomalous data also showed clustering trends corresponding to their defect class in the latent space learned with SSAD.

## 4. Discussion

### 4.1. Effect of the Principal Components

**Figure 8:**
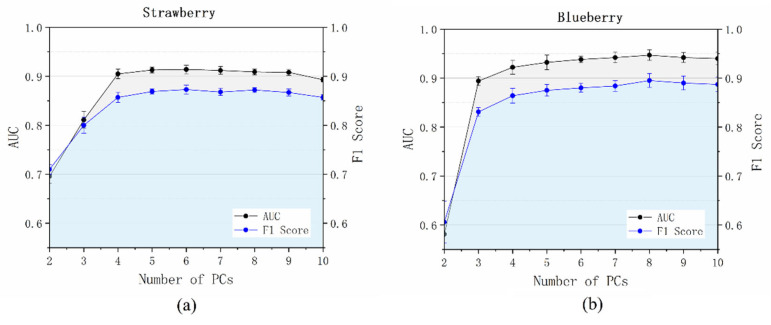
AUC and F1 score results as a function of the PC number used for building the auxiliary classifier: (**a**) strawberry data set; (**b**) blueberry data set.

In the SSAD, the number of PC images (m) used for classification should be considered a hyperparameter. In the original model, we used a five-classes classifier (*m* = 5). To investigate the impact of this hyperparameter, we changed the number of PC images *m* and built corresponding *m*-class self-supervised classifiers for anomaly detection. The obtained AUC and F1 score results are summarized in Figure 8. For both studied data sets, the AUC and F1 score first increased with the increase in PC number and then began to decrease. The best AUC of the strawberry data set was obtained with six PCs (0.914 ± 0.009), while the best performance of the blueberry data set was obtained with eight PCs (0.947 ± 0.011). Fortunately, the choice of this hyperparameter was not very sensitive: the model performance began to stabilize for an *m* greater than four for both data sets. This was in good agreement with the explained ratio of the PCs (see Figure S3); PCs with a variable explained ratio smaller than 1% make little contribution for anomaly detection.

When compared with the traditional supervised approaches for detecting the defects of strawberries [12,37,38] and blueberries [15,39], the proposed SSAD could achieve competitive results for classifying healthy and defected samples. Many of these supervised approaches focused on finding effective wavelengths corresponding to the target defects, which is not practical for anomaly detection because the potential defect type is unknown. Therefore, using the first *m* PC to characterize all wavelengths is more suitable for the ‘non-target detection’.

### 4.2. Effect of the Layers for Feature Extraction

**Figure 9:**
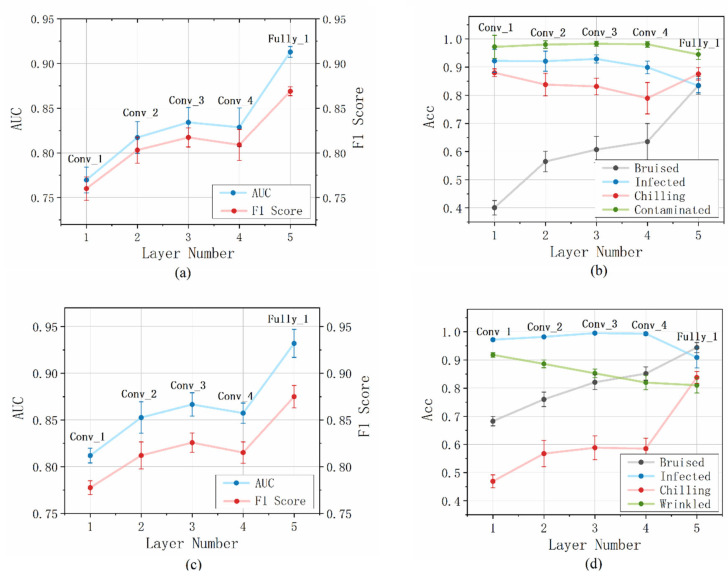
Anomaly detection results obtained with the features of different layers: (**a**) AUC and F1 results for the strawberry data set; (**b**) Acc results of each anomalous class for the strawberry data set; (**c**) AUC and F1 results for the blueberry data set; (**d**) Acc results of each anomalous class for the blueberry data set.

The self-supervised classifier extracted multi-level features of the PC images by stacking the convolution layers and a fully connected layer. In fact, each layer of the classifier can be considered a feature extractor. Here, we report the anomaly detecting results obtained with the features extracted from different layers of the PC image classifier. Figure 9a,c demonstrates the AUC and F1 score of the strawberry and blueberry data sets; the AUC increased with deeper layers in general, and the feature of the dense layer showed obvious superiority over the convolutional features. Figure 9b,d summarizes the accuracy changes in each defect class obtained with the strawberry and blueberry data sets, respectively. Different from the overall AUC, the detection accuracy of each defect class showed different trends with the number of network layers. While some of the defect classes could achieve a high detection accuracy with the first convolution layer that decreased with deeper layers, the accuracy of other defects increased gradually with the deepening of the network. In general, the shallow layers demonstrated unbalanced results for different defects, while the final fully connected layer achieved satisfactory performance for all the studied defect classes (accuracy over 80%).

### 4.3. Effect of Data Pollution

**Figure 10:**
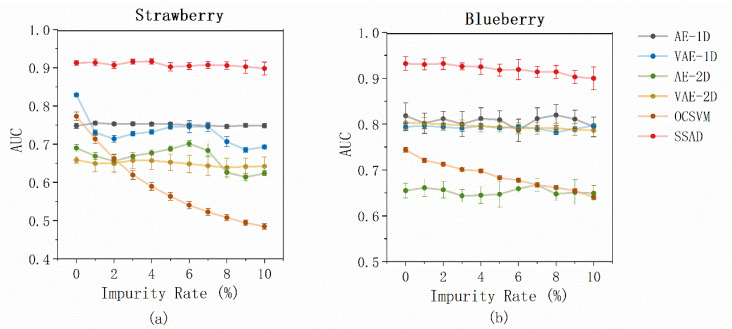
AUC results as a function of the pollution rate in the training set. (**a**) strawberry data set; (**b**) blueberry data set.

In this section, the robustness of the SSAD was validated by conducting a training data pollution experiment. The robustness against impure data is of great importance because the collected unlabeled samples often contain a small percentage of anomalies in real-world applications. A good anomaly detector should have the ability to maintain its performance against polluted data. In the data pollution experiment, a portion of the anomalous samples was randomly selected and assigned to the training set based on the polluted rate P%. As the AUC results show in Figure 10, it is not surprising that the performance of SSAD degraded with the increase in P: the AUC decreased gently from 0.913 ± 0.006 to 0.898 ± 0.017 for the strawberry data set, and it changed from 0.932 ± 0.015 to 0.900 ± 0.025 for the blueberry data set. Nevertheless, the SSAD still achieved the best performance at all polluted levels when compared with the baseline methods. In terms of the baseline methods, most of the autoencoder-based methods demonstrated relatively stable performance, whereas the OCSVM showed high requirements for sample purity, and significant deterioration was observed. This was in good agreement with the results of previous studies [21], which reported that polluted samples might easily cause the wrong hypersphere of OCSVM.

## 5. Conclusions and Future Work

This paper proposes a self-supervised anomaly detection method named SSAD for fruit defect detection based on hyperspectral imaging. In the proposed framework, an auxiliary classifier was trained with the normal samples to extract efficient ‘spectral–spatial’ features of the hyperspectral data, and then the feature similarity between the test data and the training data was utilized for anomaly evaluation. The effectiveness and versatility of SSAD was validated on two fruit data sets, namely strawberry and blueberry, each containing four fruit quality defects as anomalies. The obtained AUC results of the strawberry and blueberry data sets achieved 0.913 ± 0.006 and 0.932 ± 0.015, respectively. The overall experimental results showed that SSAD yielded superior anomaly detection performance over the comparison methods, including OCSVM, AE-1D, VAE-1D, AE-2D, and VAE-2D. The visualization results of the anomaly score distribution and t-SNE feature also demonstrate that the proposed algorithm with self-supervised classifier can effectively extract ‘spectral–spatial’ features for distinguishing normal and anomalous data. Moreover, in the data pollution experiment, SSAD demonstrated good robustness against anomalies data in the training set and outperformed all the comparison methods at all data pollution levels.

In conclusion, the SSAD is a significant improvement over the state-of-the-art methods for anomaly detection of fruits. However, as we know, PCA transformation may cause loss of spectral information to some degree. Therefore, self-supervised architecture that can process PC images and mean spectra simultaneously might yield a better performance and should be investigated in future work.

## Figures and Tables

**Table 1:** Sample information for the strawberry and the blueberry data sets.

Strawberry							
	Total	Normal		Anomaly			
		Training	Test	Bruised	Infected	Chilling	Contaminated
Number of samples	1045	300	301	139	100	105	100
Blueberry							
	Total	Normal		Anomaly			
		Training	Test	Bruised	Infected	Chilling	Wrinkled
Number of samples	1335	404	404	120	120	150	137

**Table 2:** Detailed network information of the auxiliary PC classifier in SSAD for the strawberry data set.

Strawberry Data Set		
Name of Layers	Network Parameters	Output Feature Size
Convolution_1	Filters = 16, Filter_size = 3 × 3, Activation = ReLu	120 × 120 × 16
Average_pooling_1	Filter_size = 2, Stride =2	60 × 60 × 16
Convolution_2	Filters = 16, Filter_size = 3 × 3, Activation = ReLu	60 × 60 × 16
Average_pooling_2	Filter_size = 2, Stride =2	30 × 30 × 16
Convolution_3	Filters = 16, Filter_size = 3 × 3, Activation = ReLu	30 × 30 × 16
Average_pooling_3	Filter_size = 2, Stride =2	15 × 15 × 16
Convolution_4	Filters = 4, Filter_size = 3 × 3, Activation = ReLu	15 × 15 × 4
Fully_connected_1	Nodes = 16, Activation = Tanh	16
Output	Nodes = 5, Activation = Softmax	5

**Table 3:** Detailed network information of the auxiliary PC classifier in SSAD for the blueberry data set.

Blueberry Data Set		
Name of Layers	Network Parameters	Output Feature Size
Convolution_1	Filters = 16, Filter_size = 3 × 3, Activation = ReLu	60 × 60 × 16
Average_pooling_1	Filter_size = 2, Stride =2	30 × 30 × 16
Convolution_2	Filters = 16, Filter_size = 3 × 3, Activation = ReLu	30 × 30 × 16
Average_pooling_2	Filter_size = 2, Stride =2	15 × 15 × 16
Convolution_3	Filters = 16, Filter_size = 3 × 3, Activation = ReLu	15 × 15 × 16
Convolution_4	Filters = 4, Filter_size = 3 × 3, Activation = ReLu	15 × 15 × 4
Fully_connected_1	Nodes = 16, Activation = Tanh	16
Output	Nodes = 5, Activation = Softmax	5

**Table 4:** Anomaly detection results of the strawberry data set ^1^.

Methods	AUC	F_1_ Score	Acc_Normal	Acc_Bruised	Acc_Infected	Acc_Chilling	Acc_Contaminated
OCSVM	0.773 ± 0.009	0.758 ± 0.007	0.643 ± 0.009	0.788 ± 0.020	0.594 ± 0.015	0.904 ± 0.005	0.776 ± 0.003
AE-1D	0.748 ± 0.005	0.727 ± 0.005	0.597 ± 0.007	0.684 ± 0.007	0.552 ± 0.009	**0.995 ± 0.001**	0.902 ± 0.005
VAE-1D	0.829 ± 0.004	0.784 ± 0.005	0.681 ± 0.008	0.742 ± 0.012	0.496 ± 0.016	0.753 ± 0.018	0.869 ± 0.015
AE-2D	0.690 ± 0.024	0.690 ± 0.017	0.543 ± 0.026	0.460 ± 0.051	0.764 ± 0.013	0.949 ± 0.025	0.866 ± 0.021
VAE-2D	0.659 ± 0.021	0.704 ± 0.014	0.542 ± 0.022	0.475 ± 0.014	0.767 ± 0.021	0.914 ± 0.003	0.717 ± 0.015
SSAD	**0.913 ± 0.006**	**0.869 ± 0.005**	**0.807 ± 0.007**	**0.835 ± 0.031**	**0.834 ± 0.025**	0.876 ± 0.022	**0.945 ± 0.018**

^1^ The results are presented in the form of average and 95% confidence intervals of ten resampling runs. The bold numbers represent the best performing method for each metric.

**Table 5:** Anomaly detection results of the blueberry data set ^1^.

Methods	AUC	F_1_ Score	Acc_Normal	Acc_Bruised	Acc_Infected	Acc_Chilling	Acc_Wrinkled
OCSVM	0.744 ± 0.005	0.710 ± 0.005	0.622 ± 0.007	0.789 ± 0.010	0.582 ± 0.013	**0.900 ± 0.006**	0.614 ± 0.013
AE-1D	0.818 ± 0.028	0.779 ± 0.025	0.712 ± 0.033	0.838 ± 0.044	0.769 ± 0.057	0.743 ± 0.035	0.772 ± 0.027
VAE-1D	0.794 ± 0.008	0.754 ± 0.009	0.678 ± 0.011	0.910 ± 0.014	0.630 ± 0.020	0.823 ± 0.005	0.691 ± 0.013
AE-2D	0.655 ± 0.016	0.643 ± 0.011	0.534 ± 0.015	0.699 ± 0.037	**0.991 ± 0.002**	0.602 ± 0.031	0.249 ± 0.023
VAE-2D	0.803 ± 0.005	0.768 ± 0.003	0.697 ± 0.004	0.864 ± 0.006	0.963 ± 0.003	0.420 ± 0.006	0.774 ± 0.007
SSAD	**0.932 ± 0.015**	**0.875 ± 0.012**	**0.837 ± 0.016**	**0.944 ± 0.018**	0.909 ± 0.037	0.838 ± 0.021	**0.810 ± 0.027**

^1^ The results are presented in the form of average and 95% confidence intervals of ten resampling runs. The bold numbers represent the best performing method for each metric.

## Data Availability

The datasets generated for this study are available on request to the corresponding author.

## Supplementary Materials

The following supporting information can be downloaded at: https://www.mdpi.com/article/10.3390/foods12142669/s1, Figure S1: Spectral profiles of samples in different anomalous classes: (a) strawberry data set; (b) blueberry data set. Gray lines: mean spectral curves, colored areas: spectral profiles of all the samples; Figure S2: Images of PC6 to PC10 for the strawberry and blueberry data sets; Figure S3: The variable explained ratios of the principal components: (a) strawberry data set; (b) blueberry data set; Table S1: Detailed structural parameters of the AE-1D; Table S2: Detailed structural parameters of the AE-2D; Table S3: Detailed structural parameters of the VAE-2D; Table S4: The testing time per sample obtained by different methods.
