# Supplementary material for: A Self-Supervised Anomaly Detector of Fruits Based on Hyperspectral Imaging
Source: Foods. 2023 Jul 11;12(14):2669. doi: 10.3390/foods12142669 (PMC10378827; doi:10.3390/foods12142669)
Supplement: Supplementary file 1 [file foods-12-02669-s001.zip › foods-2483659-supplementary.pdf]

Table S1. Detailed structural parameters of the AE-1D.

AE-1D (Strawberry dataset)		AE-1D (Blueberry dataset)	
Layers	Parameters	Layers	Parameters
Input	Features=181	Input	Features=181
Fully_connected_1	Nodes=32, Activation=ReLu	Fully_connected_1	Nodes=32, Activation=ReLu
Fully_connected _2	Nodes=16, Activation=ReLu	Fully_connected _2	Nodes=32, Activation=ReLu
Fully_connected _3	Nodes=32, Activation=ReLu	Fully_connected _3	Nodes=16, Activation=ReLu
Output	Nodes=181, Activation=Sigmoid	Fully_connected _4	Nodes=32, Activation=ReLu
		Fully_connected _5	Nodes=32, Activation=ReLu
		Output	Nodes=181, Activation=Sigmoid

Table S2. Detailed structural parameters of the AE-2D.

AE-2D (Strawberry dataset)		AE-2D (Blueberry dataset)	
Layers	Parameters	Layers	Parameters
Input	120×120×5	Input	60×60×5
Conv2D _1	64, 3×3, Strids=2×2, ReLu	Conv2D _1	64, 3×3, Strids=2×2, ReLu
Conv2D _2	64, 3×3, Strids=2×2, ReLu	Conv2D _2	64, 3×3, Strids=2×2, ReLu
Conv2D _3	64, 3×3, Strids=2×2, ReLu	Trans Conv2D _1	64, 3×3, Strids=2×2, ReLu
Trans Conv2D _1	64, 3×3, Strids=2×2, ReLu	Trans Conv2D _2	64, 3×3, Strids=2×2, ReLu
Trans Conv2D _2	64, 3×3, Strids=2×2, ReLu	Trans Conv2D _4	5, 3×3, Strids=1×1
Trans Conv2D _3	64, 3×3, Strids=2×2, ReLu		
Trans Conv2D _4	5, 3×3, Strids=1×1		

Table S3. Detailed structural parameters of the VAE-2D.

VAE-2D (Strawberry dataset)		VAE-2D (Blueberry dataset)	
Layers	Parameters	Layers	Parameters
Input	120×120×5	Input	60×60×5
Conv2D _1	64, 3×3, Strids=2×2, ReLu	Conv2D _1	64, 3×3, Strids=2×2, ReLu
Conv2D _2	64, 3×3, Strids=2×2, ReLu	Conv2D _2	64, 3×3, Strids=2×2, ReLu
Conv2D _3	64, 3×3, Strids=2×2, ReLu	FC_1 (z_mean)	Nodes=64
FC_1 (z_mean)	Nodes=256	FC_2 (z_std)	Nodes=64
FC_2 (z_std)	Nodes=256	FC_3	Nodes=15*15*2, ReLu
FC_3	Nodes=15*15*4, ReLu	Trans Conv2D _1	64, 3×3, Strids=2×2, ReLu
Trans Conv2D _1	64, 3×3, Strids=2×2, ReLu	Trans Conv2D _2	64, 3×3, Strids=2×2, ReLu
Trans Conv2D _2	64, 3×3, Strids=2×2, ReLu	Trans Conv2D _4	5, 3×3, Strids=1×1
Trans Conv2D _3	64, 3×3, Strids=2×2, ReLu		
Trans Conv2D _4	5, 3×3, Strids=1×1		

Table S4. The testing time per sample obtained by different methods.

	OCSVM	AE-1D	VAE-1D	AE-2D	VAE-2D	SSAD
Testing time per sample (ms)	0.015	4.341	27.295	4.665	28.779	0.027

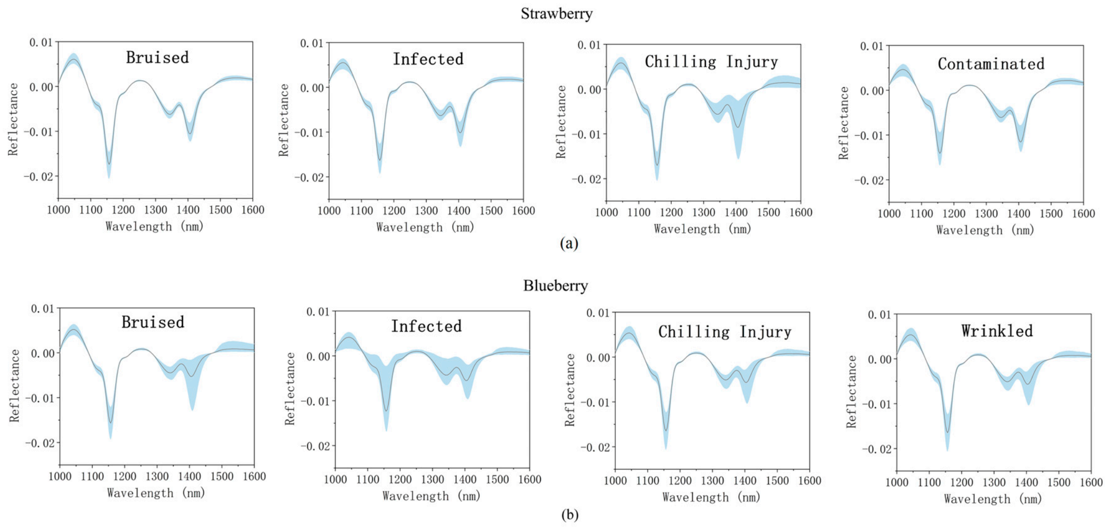

Figure S1. Spectral profiles of samples in different anomalous classes: (a) strawberry data set; (b) blueberry data set. Gray lines: mean spectral curves, colored areas: spectral profiles of all the samples.

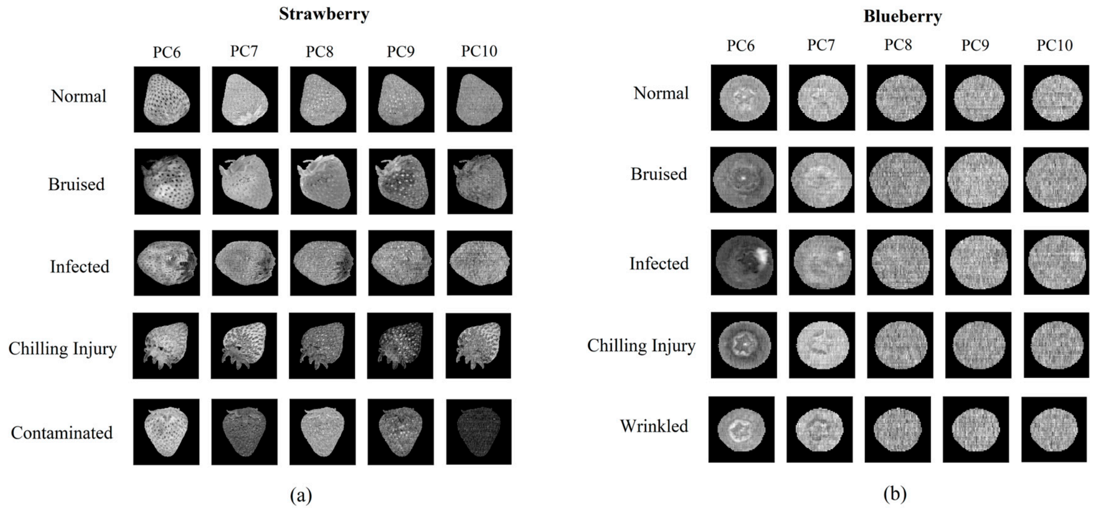

Figure S2. Images of PC6 to PC10 for the strawberry and blueberry data sets.

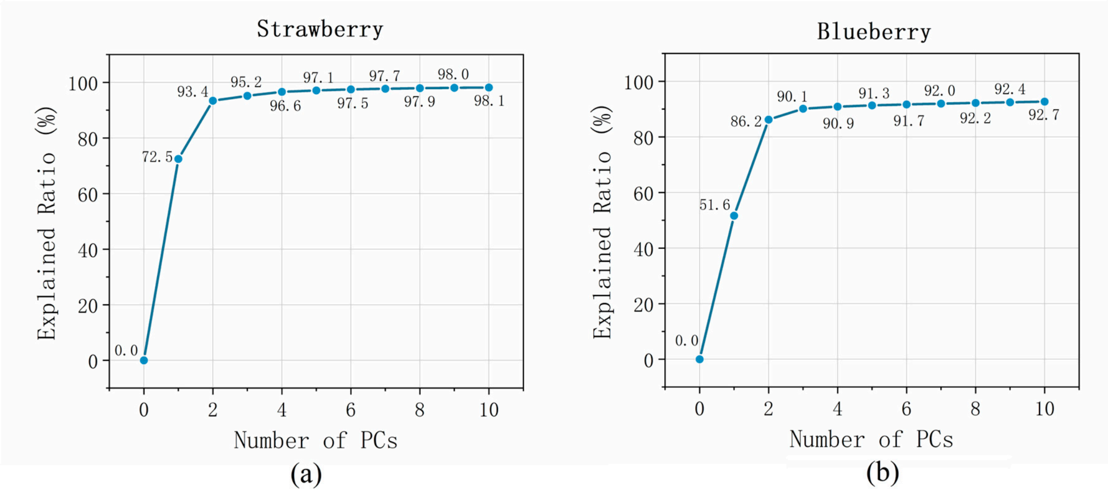

(a) (b)

Figure S3. The variable explained ratios of the principal components: (a) strawberry data set; (b) blueberry data set.
